# The Impact of Mother’s Living Environment Exposure on Genome Damage, Immunological Status, and Sex Hormone Levels in Newborns

**DOI:** 10.3390/ijerph17103402

**Published:** 2020-05-13

**Authors:** Aleksandra Fucic, Mirta Starcevic, Nada Sindicic Dessardo, Drago Batinic, Sasa Kralik, Jure Krasic, Nino Sincic, Damir Loncarevic, Vedrana Guszak

**Affiliations:** 1 Institute for Medical Research and Occupational Health, 10000 Zagreb, Croatia; 2 Scientific Center of Excellence for Reproductive and Regenerative Medicine (CERRM), 10000 Zagreb, Croatia; mirta_ps@yahoo.com (M.S.); sindin2@net.hr (N.S.D.); drago.batinic@mef.hr (D.B.); skralik@kbc-zagreb.hr (S.K.); jure.krasic@mef.hr (J.K.); nino.sincic@mef.hr (N.S.); vguszak@yahoo.com (V.G.); 3 University Hospital Center Zagreb, 10000 Zagreb, Croatia; loncarevic.dr@gmail.com; 4 Medical School, University of Zagreb, 10000 Zagreb, Croatia

**Keywords:** estradiol, testosterone, endocrine disruptor, newborn, genome damage, diet, IL-6, biomonitoring

## Abstract

*Background:* The aim of this study was to compare for the first time IL-6 (Interleukin 6), testosterone (T) and estradiol (E) levels, their ratio (E/T), micronucleus (MN), and nuclear bridge (NB) frequency between newborns with regard to their mother’s residency and diet. Our results should enable an assessment of the possible environmental endocrine effects and interaction between biomarkers, pointing to possible associated health risks. *Methods:* Fifty full-term newborns of both sexes, whose mothers were healthy and not occupationally exposed to any known carcinogen, were analyzed. All of the mothers filled in a detailed questionnaire. *Results:* The results showed significantly higher levels of E in newborns of mothers with agricultural residency than those born by mothers with urban residency. Significantly, lower levels of E were measured in newborns of mothers who drank milk and carbonated beverages more frequently. Testosterone was significantly higher in boys of mothers with agricultural residency than from mothers with urban residency. Residence and other parameters had no impact on the difference in MN frequency. IL-6 levels were higher in newborns of mothers with agricultural residency. NB levels were significantly associated with E. A significant association between E levels and IL-6 was found. *Conclusion:* Our results were the first to show a significant impact of the mother’s agricultural residency and diet on their newborns’ sex hormone and IL-6 levels and their association.

## 1. Introduction

The complex mechanisms and dynamics of intrauterine development, which, if disturbed, may have lifelong consequences on health and susceptibility to xenobiotics, represent the top priorities of research. Of specific interests are endocrine disruptors (EDS), which may interact with a large number of mechanisms in the human body that cause not just traditionally sex hormone-related diseases but are also involved in mechanisms of carcinogenesis, immunology disturbance, infertility, and brain development [1].

A large number of contaminants in the air, food, and water cross the placental barrier. Although exposure to urban pollution has been for decades associated with a large number of diseases, an agricultural living environment may also be associated with exposure to different xenobiotics, especially pesticides [2,3]. Endocrine mechanisms by which pesticides affect human health are broadly investigated; however, some groups, such as fungicides, which act as aromatase inhibitors, demand more attention [4,5]. Levels of pesticides in the placenta and cord blood suggest that both diffusion and active transport are involved in their transfer from mother to fetus [6,7]. A recent study shows that testosterone and estradiol levels are inversely associated with organophosphate pesticides in cord blood [8].

Diet, smoking, and residency of the mother are reflected in genome damage in newborns [9,10,11]. Micronucleus frequency (MN) by which clastogenic and aneugenic mechanisms of xenobiotics are detected is a reliable method for estimation of cancer risk on a group level and has been successfully applied in biomonitoring of newborns for decades [9,12,13]. However, most diseases that may have originated in transplacental exposures are based on multi-scale disturbances. That means genome damage should be included in biomonitoring data interpretation as a segment and not a single axis of health risk estimation. For example, the impact of in utero exposure to dioxin-like chemicals has been shown to cause both increased MN frequency and sex hormone or sex hormone receptor disturbances [14,15].

Early life disturbances in estradiol (E), testosterone (T) levels, and their ratios have been shown to be important not only in different infertility problems, such as polycystic ovarian or low sperm quality, but also in immunological and inflammatory mechanisms with lifelong health effects [16,17,18,19]. Thus, higher levels of neonatal androgens are associated with increased testicular cancer risk [20]. It should be mentioned, however, that lifestyle and diet during adulthood might also contribute to infertility problems [21,22].

Sex-specific health risks associated with transplacental environmental stressors are recognized, but still, the much more integrative investigation is needed. It is known that transplacental exposure of boys has shown significantly more disturbance of fetal growth than girls [23,24,25,26].

Disturbances of cytokine levels in newborns are reported to be associated with transplacental exposure due to different environmental settings of the mother [27,28]. IL-6, as a pro-inflammatory cytokine, is involved in a number of physiological processes and chronic diseases, and if disturbed in early life, it may cause neurobehavioral or immune responses changes [29,30,31]. The interaction between sex hormones and IL-6 has been described in cancer promotion [32,33], but there is no data on such interaction in newborns, especially in cases when exposure to xenoestrogens is suspected.

The aim of the current study was to find an association between mothers’ living environment and the level of MN, nuclear bridge (NB) frequency, estradiol, testosterone levels, the ratio between E and T, and IL-6 levels in their newborns. Parameters, such as smoking, place of residence, and newborn’s gender, were selected as the most important based on the hypothesis of their association with applied biomarkers. This was the first study ever to compare genome damage, cytokine, and sex hormones levels in cord blood with the mother’s environmental exposure.

## 2. Materials and Subjects

Fifty healthy mothers who gave birth to full-term newborns were informed about the study and, after signing a written consent, filled-in detailed questionnaires on their medical and family history, occupational exposures, diet (meat, soft beverages, alcohol, dairy products, vegetables, fruit), hobbies (plastic materials, paint, glue), residence, in-house environment during pregnancy (renovation), and smoking. In addition to the information collected in the questionnaire, the residence was defined by Google Earth. Urban residence was defined in case that residence was next to the highway (up to 50 m) or within the Zagreb city district. The agricultural residence was defined if the residence was next to wheat, corn vegetable, vineyard, or orchard field (50 m). The mothers with the help of a neonatologist filled in the questionnaires in order to avoid any unclear questions. Newborns with malformations were excluded from the study. The exclusion criteria were parental occupational exposure to chemical agents or radiation, parental chemotherapy or radiotherapy during life, parental addiction to drugs of abuse and alcohol, and malformations in the newborn or siblings. The questionnaire was based on experiences from the NewGeneris project [13] adjusted for Croatian lifestyle specificities.

Cord blood samples were collected within a 10-month period. Samples were frozen at −80 °C for sex hormones and IL-6 analyses within a few hours. Cell culture for MN assay was launched within 24 h after delivery.

The Ethics Committee of the University Hospital Center “Zagreb”, Zagreb, Croatia, approved the study (021-1/41-18).

### 2.1. Micronucleus Assay

Umbilical cord blood was collected in heparinized and EDTA tubes (BD Vacutainer, Plymouth, UK) immediately after delivery. For micronucleus assay, heparinized blood was used. The cell culture for MN assay was initiated by phytohemagglutinin (Remel, UK) within 24 h after delivery. Umbilical cord blood was diluted by RPMI 1640 (Sigma Aldrich, St. Louis, MN, USA) 1:3. Cultivation was carried at 37 °C (5% CO_2_ in an incubator) in tissue culture flask (TPP, Trasadingen, Switzerland) with a total culture volume of 7.9 mL. After 44 h, cytochalasin B (Sigma Aldrich, St. Louis, MN, USA) was added at a final concentration of 6 µl/mL. At 72 h, cell cultures were harvested, centrifuged, and 90 mM KCl hypotonic solution (Merck, Germany) was added at 4 °C. After 15 min, samples were centrifuged and fixed in methanol:acetic acid 3:1 twice. The suspension was dropped on clean slides and stained by Giemsa (Merck, Germany). Duplicated cultures were prepared for each subject. Slides were double coded.

For each subject, 2000 binucleated cells were manually scored by a single scorer following the criteria of the Human MicroNucleus project. Additionally to MN frequency, nuclear bridges were scored [34].

### 2.2. Sex Hormones

Estradiol and testosterone were determined in the serum using the commercial Chemiluminescent Microparticle Immunoassay kits (Abbott, Chicago, IL, USA) for the quantitative determination of testosterone (2P13) and estradiol (Abbott, Architect i1000sr, Chicago, IL, USA).

### 2.3. Interleukin 6 (IL-6)

The serum concentration of IL-6 was measured by human IL-6 sandwich enzyme immunoassay according to the manufacturer (Quantikine^®^ELISA, Catalog Number D6050, R&D Systems Inc., Minneapolis, MN, USA). Briefly, serum samples and standards were added to microplate wells coated with a monoclonal antibody specific for human IL-6. After washing away any unbound substances, an enzyme-linked polyclonal antibody specific for human IL-6 was added to the wells. Following a wash to remove any unbound antibody-enzyme reagent, a substrate solution was added to the wells, and color developed in proportion to the amount of IL-6 bound in the initial step. The color development was stopped, and the intensity of the color was measured.

### 2.4. Statistics

For numerical variables, normality of data distributions was tested using the Shapiro–Wilk test. Variables were described by means and standard deviations if their distributions were normal and by medians and interquartile ranges otherwise. For categorical variables, the number of respondents and the percentage of the study population belonging to each category were shown.

Differences between groups of respondents for normally distributed variables were analyzed using a t-test. For other numerical variables, the Mann–Whitney test was used. For descriptive analysis and tests of differences between groups, values of IL-6 below limit of detection (LOD) were assigned values of 0.5 LOD (LOD = 0.7).

To assess associations between mothers’ age, residency, smoking, dietary habits, biocides and disinfectants usage, and wall painting/decorating, on the one hand, and hormone levels, IL-6, and markers of genome damage, on the other, multiple regression models were used.

All regression models were controlled for the mother’s place of residence, smoking status, and newborn’s gender. In addition to these three confounders, only one independent variable was entered at a time in each regression model due to the small sample size. To control for multiple comparisons, Bonferroni correction was applied to models corresponding to the same dependent variable (11 comparisons for each dependent variable; the level of significance set to 0.0045). In the case of testosterone and E/T ratio, where regression models were separated by the newborns’ sex, this correction was applied to models for each sex separately.

Associations with estradiol, testosterone, E/T ratio, and nuclear division index (NDI) as dependent variables were analyzed in linear regression models. In order to approach the normality of residuals, these dependent variables were log-transformed prior to the analyses. For all other dependent variables, logistic regression models were used. For logistic regression models, dependent variables were dichotomized as follows: IL-6—above LOD vs. below LOD; MN/1000—greater than 2 vs. 0–2; bridge/1000—greater than 0 vs. 0; bud/1000—greater than 0 vs. 0. Since only two mothers with rural residence had IL-6 values below LOD, in this case, Firth’s correction for the logistic regression model was applied.

Due to the small number of mothers reporting alcohol consumption (*N* = 3), this variable was not used as a predictor in regression models. However, as a sensitivity analysis, results from regression models with statistically significant associations were compared to the results of models with the same variables on the subsample of mothers reporting no alcohol consumption.

Associations between dependent variables (hormone levels, IL-6, and markers of genome damage) were assessed using Spearman’s correlation.

All statistical analyses were conducted using statistical programs STATA, version 14.2 (StataCorp, College Station, TX, USA) and R version 3.5.0 (R Foundation for Statistical Computing, Vienna, Austria).

## 3. Results

In the study, cord blood samples of 50 full-term newborns of both sexes (25 girls) were analyzed for E, T, IL-6 levels, MN, and NB frequency and associated with different exposure factors during pregnancy. All mothers were healthy during pregnancy and at the time of delivery. In total, 75% of newborns were born vaginally.

A detailed questionnaire was used for data collected on mother occupational and environmental exposure, age, weight gain during pregnancy, delivery type, residency, diet, smoking and drinking habit, biocide usage, and wall painting/decorating. The residency was defined by the mother’s address during the pregnancy as agricultural in the case when the residence was surrounded with agriculturally-used soil during pregnancy. All mothers consumed vegetables and fruits more than three times per week, and therefore, these parameters were not included in the analysis.

The mean age of mothers was 32.10 ± 5.40 (urban residency 32.64 ± 5.34; agricultural residency 31.06 ± 5.53; *t*-test, *p* = 0.341). The mean body mass gain for mothers was 13.80 ± 4.84 kg (urban residency 14.42 ± 4.46 kg; agricultural residency 12.59 ± 5.46 kg; *t*-test, *p* = 0.242).

Table 1 shows the descriptive data of the studied group. Boys and girls did not differ by body mass. Results of the analysis showed that boys had significantly higher levels of testosterone than girls (1.078 (0.963–1.346) vs. 0.862 (0.695–1.058), respectively), and boys had significantly lower MN frequency (4 (2–4) vs. 4 (4–8)/1000 cells, respectively) (Table 1).

With respect to the type of residency (Table 2), E levels were significantly lower in the urban subsample (2214 (881–2684) pg/mL vs. 3231 (2405–4771) pg/mL in rural subsample), as well as E/T ratio (2511 (1128–2946) vs. 3277 (2250–4186)) and IL-6 levels (<LOD (<LOD–3.88) vs. 6.38 (4.74–9.50)). No other differences in hormone levels and markers of genome damage were noted between these two groups of respondents.

Associations that were statistically significant in multivariate linear regression models (dependent variables: estradiol, testosterone, E/T ratio) are shown in Table 3. Values of T in male newborns were significantly higher than values in female newborns even after controlling for the place of residence and cigarette smoking and applying Bonferroni’s correction. Similarly, values of estradiol remained significantly lower in the urban subsample even after controlling for cigarette smoking and newborn’s sex. Additionally, lower values of testosterone were associated with urban residency, but only for male newborns (1.01 (0.92–1.15) ng/mL vs. 1.41 (1.17–2.35) ng/mL for an urban and rural agricultural subsample, respectively). Lower values of estradiol were associated with more frequent milk consumption (estradiol 3635 (2981–4187) pg/mL vs. 2192 (886–2818) pg/mL for less and more frequent consumption, respectively) and carbonated beverages (2716 (2122–3844) pg/mL vs. 881 (754–1879) pg/mL for less and more frequent consumption, respectively). While testosterone levels were somewhat higher in male newborns of mothers with more frequent beef or pork intake (0.999 (0.892–1.081) ng/mL vs. 1.182 (0.994–1.412) ng/mL for less and more frequent consumption, respectively), this difference was not statistically significant after applying Bonferroni’s correction. The E/T ratio was not significantly associated with any of the considered predictors in multiple linear regression models.

Associations that were statistically significant in multivariate logistic regression models (dependent variables: IL-6, MN, bridge, bud) are shown in Table 4. Levels of IL-6 remained statistically higher in the agricultural residency subsample even after controlling for cigarette smoking and newborns’ sex. While higher IL-6 values were noted for male newborns and mothers that consumed coffee more frequently (<LOD (<LOD–3.69) vs. 3.82 (<LOD–6.52) for less and more frequent consumption, respectively), these associations were not statistically significant after applying Bonferroni’s correction. MN, bridge, and bud frequencies were not significantly associated with any of the considered predictors in multiple logistic regression models.

All associations that were statistically significant in multiple regression models with Bonferroni’s correction remained significant after excluding three mothers who reported alcohol consumption, except for association of milk consumption and estradiol (*p* = 0.012 for mothers who did not report alcohol consumption; statistically significant only if Bonferroni’s correction was not applied).

When association of the dependent variables was assessed, a significant association was found for E and E/T ratio (R = 0.86, *p* < 0.001); E and T (R = 0.41, *p* = 0.003); E and IL-6 (R = 0.29, *p* = 0.045); E and NB frequency (R = −0.31, *p* = 0.029); for E/T ratio and NB frequency (R = −0.30, *p* = 0.032).

## 4. Discussion

The results of this study showed, for the first time, the impact of mother’s residence and diet on E, T, and IL-6 levels in their healthy full-term newborns. Testosterone levels in male newborns were significantly higher than values in female newborns. Levels of E and IL-6 were significantly higher in newborns of mothers with agricultural residency in comparison with newborns of mothers with urban residency. Significantly, higher levels of T in newborns were associated with the agricultural residency of their mothers in comparison with newborns of mothers with urban residency, but only for male newborns. Significantly lower levels of E in newborns were associated with mother’s more frequent milk and carbonated beverage intake. In male newborns of mothers who consumed dark meat, more than three times per week, there was a significant increase in T levels, although this difference was statistically significant only before adjustment for multiple testing. A significant association was found for E and E/T ratio, E and T, E and IL-6, E and NB frequency, and for E/T ratio and NB frequency.

Knowledge of the impact of sex hormone disturbances on health risks has been shifting, during the last few decades, from adults to pubertal, prepubertal, and newborn population as data are accumulated [2,35]. According to the best of our knowledge, in our study, we, for the first time, showed that at birth, male and female newborns significantly differed in testosterone levels and MN frequency. Although this might be a transient phenomenon, such results are a valuable contribution to future investigations into transplacental xenobiotic effects.

Data on the E and T levels in newborns of mothers who are environmentally exposed to the agricultural living environment are anecdotal [36,37]. Our results were the first to show significantly higher E levels in newborns of both sexes and significantly higher levels of T in male newborns born by mothers with agricultural than by mothers with urban residences. As shown in an animal model, increased E levels in newborns associated with elevated total cholesterol and low-density lipoprotein cholesterol suggest possible metabolic disorder later in life [38]. It is known that a number of pesticides are endocrine disruptors [EDCs), of which some couple with estrogen and androgen receptors, while others, such as fungicides, are aromatase inhibitors and increase T levels [39,40]. Whether such results are the consequence of aromatase disturbances or the coupling of EDCs agents with estrogen receptors causing some kind of feedback mechanisms should be answered in the future. However, the detected disturbances in sex hormones may be an underlying mechanism that could explain lower male fertility and increased testicular cancer incidence in men with agricultural residency [41,42,43,44].

The significantly lower levels of E detected in our study in newborns whose mothers consumed carbonated beverages during pregnancy at least once per day could be suggested to be associated with an intake of phthalates emitted from polyethylene terephthalate (PET) bottles [45], although phthalate metabolites were not measured in this study. The levels of phthalates in PET bottles significantly depend on the duration and conditions of storage [46], about which consumers are not aware of. Although phthalate levels in some studies are below 6 μg·L^−1^, which is the maximum admissible concentration in water recommended by the U.S. Environmental Protection Agency, it is stressed that these levels are safe for adult populations only [47]. Similarly, significantly lower levels of E were detected in newborns whose mothers drank one glass of milk per day during pregnancy. Several studies have confirmed that estrogen levels in milk and dairy products are a significant source of estrogen due to the milking of pregnant cows, which may cause health risks [48,49]. Decreased levels of E in our study could be a consequence of the mechanisms in which xenoestrogen or estrogen of animal origin couples to estrogen receptors during intrauterine development, which, in turn, reduces the production of intrinsic estrogen, as shown in an animal model by the phytoestrogen quercetin [50,51].

Significantly increased IL-6 levels in newborns of mothers with agricultural residency in comparison with newborns of mothers with urban residency was in concordance with only one other similar study that describes the association between pesticides and IL-6 in newborns [52]. However, an important result of our study was the significant association between E and IL-6. Such an association is significant in cases of carcinogenesis as E stimulates IL-6 expression [33,53]. The cross-talk between E and IL-6 is significant in the modulation of the immune response, but their interaction depends on the age, tissue, and sex of an organism [54,55]. In adults, E also stimulates the production of IL-6 in lymphocytes [56]. Based on these facts, it could be hypothesized that the increased IL-6 levels in our study might have been a consequence of an increased E effect and not directly the consequence of transplacental exposure to xenobiotics. The disturbance of the axis between E and IL-6 at birth might be an indicator of possible immunological misbalances as well as of the need for further biomonitoring of newborns.

Micronucleus and NB frequencies were not significantly associated with newborns with any of the considered exposure types in multiple logistic regression models in the current study. Our result regarding the MN frequency in newborns was in concordance with published data [9,57]. Such a result might have resulted from the fact that very few mothers smoked cigarettes and drank alcohol during pregnancy. It is interesting, however, that a significant association between E, E/T, and NB was detected. Nucleoplasmic bridge originates from dicentric chromosomes, telomere end fusions, or in case of defective separation of sister chromatids at anaphase [58]. In vitro experiments have shown that E has the ability to cause chromatid non-disjunction [59,60]. Future investigations will show possible associations with health risks, especially in cases of females, as early oocytes are at the diplotene stage of the meiosis I already during fetal life, during which increased E may cause lifelong consequences.

Although, apparent only, before adjustment for multiple testing, significantly increased levels of E in newborns of mothers who consumed dark meat more than three times per week deserve attention. The origin of E in newborns of mothers with such diets may be a consequence of meat contaminated by hormones in order to increase the production of muscle meat [61]. Although the levels may be relatively low, they still could represent a health risk, especially in developing organisms [62].

Our study limitation was the small number of subjects, which is not rare in the case of biomonitoring of the newborn population. However, statistical analysis and interpretation were carefully reasoned in order to achieve the reliability of results. In addition, measurements of xenobiotics in cord blood are planned to be conducted in the future. However, the significant added value of this study is the groundbreaking integration of biomarkers in order to provide insight into the mechanisms known to be critical during intrauterine development with possible lifelong health consequences.

## 5. Conclusions

In conclusion, our study confirmed the significance of sex hormones as biomarkers of transplacental exposure and their significant association, while the association with MN frequency was not detected. The need for sex-specific preventive measures and risk assessment had already been recognized. Levels of E, T, and IL-6 showed higher sensitivity as biomarkers of transplacental exposure in this study than the MN assay, pointing the significance of multiscale biomarker application in biomonitoring. The introduction of an integrated analysis of the crosstalk between genome damage, cytokine levels, and sex hormone levels might contribute to a personalized approach to a newborn just as lifelong medical care.

## Figures and Tables

**Table 1:** Newborns’ sex, weight, testosterone, estradiol, and IL-6 levels and markers of genome damage (*N* = 50).

Variables	Values	Boys	Girls	*p*-Value
Sex (female), No (%)	25 (50)			
Body mass (g), mean (SD)	3498 (419)	3528 (437)	3468 (406)	0.618
Estradiol (pg/mL), median (IQR)	2546 (1144–3654)	2405 (1879–3596)	2670 (953–3674)	0.686
Testosterone (ng/mL), median (IQR)	**0.974 (0.831–1.157)**	**1.078 (0.963–1.346)**	**0.862 (0.695–1.058)**	**0.003**
E/T ratio, median (IQR)	2661 (1251–3488)	2564 (1740–2854)	3013 (1219–3675)	0.325
MN/1000, median (IQR)	**4 (2–6)**	**4 (2–4)**	**4 (4–8)**	**0.043**
IL-6, median (IQR)	1.74 (<LOD–6.51)	3.69 (<LOD–6.51)	<LOD (<LOD –6.51)	0.494
Bridge/1000, median (IQR)	0 (0–2)	0 (0–2)	0 (0–2)	0.816
Bud/1000, median (IQR)	0 (0–0)	0 (0–0)	0 (0–2)	0.567

Significant results are presented in bold. Micronucleus (MN), estradiol (E), testosterone (T).

**Table 2:** Differences in newborns’ hormone levels, IL-6, and markers of genome damage (*N* = 50) by type of residency.

Variables	Urban (*N* = 33)	Agrar (*N* = 17)	*p*-Value
Estradiol (pg/mL), median (IQR)	**2214 (881–2684)**	**3231 (2405–4771)**	**0.009**
Testosterone (ng/mL), median (IQR)	0.974 (0.827–1.078)	1.092 (0.862–1.412)	0.264
E/T ratio, median (IQR)	**2511 (1128–2946)**	**3277 (2250–4186)**	**0.013**
MN/1000, median (IQR)	4 (2-6)	4 (2-6)	0.750
IL-6, median (IQR)	**<LOD - 3.88)**	**6.38 (4.74–9.50)**	**<0.001**
NDI, mean (SD)	2.56 (0.32)	2.61 (0.25)	0.551
NB/1000, median (IQR)	0 (0–2)	0 (0–2)	0.392
Bud/1000, median (IQR)	0 (0–0)	0 (0–2)	0.154

Significant results are presented in bold.

**Table 3:** Results of multivariate linear regression models in newborns.

	Estradiol	Estradiol	Testosterone (Whole Sample)	Testosterone (Male Newborns Only)
Intercept	**8.19**	**7.76**	0.09	−0.15
	**[7.72, 8.67]**	**[7.44, 8.07]**	[−0.06, 0.25]	[−0.34, 0.05]
	***p* < 0.001**	***p* < 0.001**	*p* = 0.236	*p* = 0.129
Agricultural residency	**0.77**	**0.77**	**0.22**	**0.45**
	**[0.35, 1.19]**	**[0.36, 1.18]**	**[0.01, 0.42]**	**[0.17, 0.73]**
	***p* = 0.001**	***p* < 0.001**	***p* = 0.040**	***p* = 0.003**
Cigarette smoking	−0.09	0.11	0.01	0.14
	[−0.63, 0.45]	[−0.42, 0.63]	[−0.26, 0.27]	[−0.19, 0.46]
	*p* = 0.747	*p* = 0.680	*p* = 0.950	*p* = 0.382
Female sex	−0.29	−0.31	**−0.33**	-
	[−0.68, 0.11]	[−0.69, 0.08]	**[−0.52, −0.13]**	
	*p* = 0.149	*p* = 0.115	***p* = 0.001**	
Consuming milk at least 1 per day	**−0.76**	**-**	**-**	**-**
	**[−1.22, −0.30]**			
	***p* = 0.002**			
Consuming carbonated beverages at least 1 per day	**-**	**−0.91**	**-**	**-**
		**[−1.41, −0.41]**		
		***p* = 0.001**		
Beef or pork intake at least 3 per week	**-**			**0.28**
				**[0.03, 0.54]**
				***p* = 0.033**
Model *p*	<0.001	<0.001	0.005	0.003
Adjusted R^2^	0.28	0.31	0.19	0.41

For each variable in the model, the results are shown as the regression coefficient [95% confidence interval] with the coefficient *p*-value. Dependent variables were log-transformed. Significant results are presented in bold.

**Table 4:** Results of the multivariate logistic regression model for IL-6 in newborns.

	IL-6 above LOD
Intercept	−1.50
	[−3.86, 0.18]
	*p* = 0.083
Agricultural residency	**38.58**
	**[6.08, 512.79]**
	***p* < 0.001**
Cigarette smoking	0.82
	[0.09, 8.09]
	*p* = 0.856
Female sex	**0.14**
	**[0.02, 0.67]**
	***p* = 0.012**
Consuming coffee at least 1 per day	**6.55**
	**[1.14, 71.44]**
	***p* = 0.034**
Model *p*	<0.001

For each variable in the model, the results are shown as odds ratio [95% confidence interval] with coefficient *p*-values. The intercept is shown in the original form. Significant results are presented in bold.
